# Prediction of energy consumption in four sectors using support vector regression optimized with genetic algorithm

**DOI:** 10.1016/j.heliyon.2025.e41765

**Published:** 2025-01-09

**Authors:** Md. Sadikul Hasan, Md. Tarequzzaman, Md. Moznuzzaman, Md Abdul Ahad Juel

**Affiliations:** a Department of Electrical and Electronic Engineering, Jashore University of Science and Technology, Jashore-7408, Bangladesh; b Department of Electrical and Electronic Engineering, Bangladesh University of Business and Technology, Dhaka-1216, Bangladesh

**Keywords:** SVR, Carbon emissions, Prediction, Energy consumption, Energy management

## Abstract

Effectively managing and optimizing energy resources to accommodate population growth while minimizing carbon emissions has become increasingly intricate. A proficient approach to this dilemma is accurately predicting energy usage and emissions across diverse sectors. This paper unveils a genetic algorithm (GA)-optimized support vector regression (SVR) model designed to (i) predict electricity generation, (ii) predict energy consumption in four primary sectors—residential, industrial, commercial, and agricultural, and (iii) estimate sector-specific carbon emissions. The proposed model's efficacy is assessed by calculating the R^2^ value, mean absolute error (MAE), root mean squared error (RMSE), and residual plot. The model achieved high accuracy in predicting energy generation, with an MAE of 1.18 %, and yielded reliable sectoral consumption predictions, reflected in MAE values of 1.22 % (residential), 4.98 % (industrial), 4.40 % (commercial), and 4.04 % (agricultural). The residuals exhibited homoscedasticity, and the R^2^ value approached one. The model predicts that by 2027, the residential sector will consume 55748.66 GWh of energy, the commercial sector 14892.49 GWh, the industrial sector 32642.35 GWh, and the agricultural sector 2288.37 GWh. It has been predicted that by 2027, these four sectors will release 75437.96-billion-gram carbon equivalents.

## Abbreviation section

GA -: Genetic Algorithm
SVR-: Support Vector Regression
MAE -: Mean Absolute Error
RMSE -: Root Mean Squared Error
ANN-: Artificial Neural Network
LSTM –: Long Short-Term Memory
ARIMA-: Auto Regressive Integrated Moving Average
SARIMA-: Seasonal Auto Regressive Integrated Moving Average

## 1 Introduction

To attain the goals of sustainable development of emerging nations in energy sectors, certain impediments must be confronted: The objectives encompass fulfilling the increasing energy requirements, advocating for a more environmentally friendly combination of fuel for generating power, mitigating the negative impacts on the environment and tackling climate change, enhancing the accessibility and affordability of energy, and ensuring the financial viability of new power generation projects through efficient governance [1]. Energy consumption is intricately linked to these challenges. Precisely assessing energy consumption may facilitate the precise determination of escalating energy demand, the fuel mix for generation, detrimental environmental impacts, energy accessibility, and cost. Since the eighth century, global energy consumption has risen at an annual pace of 2.5 %, prompting experts to contemplate the future growth of this demand [2]. Global electricity generation has increased at a rate surpassing primary energy supply; nevertheless, the need for improved generating capacity and investment varies among different economies. Emerging economies require augmented power generation to promote industrialization and provide electricity for rural areas. Developed economies require augmented power production due to the escalating use of electric appliances [3]. Prior studies have demonstrated that energy consumption in emerging nations is projected to rise by 3 % annually, while energy demand in developed countries is anticipated to grow by 0.9 % per year [4]. Industries in developing countries are projected to become the primary consumers of energy shortly [5].

This circumstance is similarly relevant to Bangladesh. Rapid industrialization, economic advancement, and rising population increase the country's power usage. Bangladesh's energy consumption spike is a direct consequence of the nation's swiftly advancing economy and rising gross domestic product (GDP). Conversely, fossil fuel reserves are inadequate to meet the rising energy demand. Bangladesh's power generation sector predominantly depends on natural gas, attributed to its substantial reserves that exceed those of alternative fossil fuel sources. Projections suggest that Bangladesh's natural gas reserves are expected to be depleted by 2028, presenting a considerable threat to the nation's future energy stability [6]. Consequently, Bangladesh must promptly amend its energy strategy to comply with future energy demands and attain sustainable economic development. Accurate prediction of energy consumption can facilitate this achievement. Forecasting energy consumption is essential for directing energy allocation [7], formulating conservation policies [8], and improving the energy infrastructure. Moreover, precise energy predictions can aid management in expediting economic development [9]. As a result, energy consumption predictions have been regarded as a critical and challenging problem in both industry and academia. The determination of this outcome is contingent upon several elements, including but not limited to GDP, population size, level of urbanization, and import and export activities [5,10]. Establishing accurate forecasts of generated electricity is the initial step toward achieving precise predictions of electrical energy consumption. Inaccurate projections of energy consumption that result in load shedding jeopardize the national grid's schedule, leading to a declining standard of living. Moreover, understanding future energy production can aid decision-makers in efficiently mitigating carbon emissions in the energy industry. Furthermore, integrating zero-carbon sources is beneficial for meeting future energy requirements. Projecting energy consumption by sector provides superior benefits than projecting overall energy consumption. Forecasting energy consumption across diverse sectors can enhance governmental efficiency and guide decision-making for energy-producing entities. Sector-specific projections can assist policymakers in making informed choices that foster societal progress toward sustainability [11]. Alongside evaluating sector-specific energy consumption, it is imperative to quantify carbon emissions, particularly from the energy sector. Researchers anticipate the Earth's surface temperature will increase by 2.1 °C by 2100. The consensus among individuals is that the principal factor contributing to the Earth's warming trend is the emission of greenhouse gases, particularly carbon dioxide (CO_2_), from power plants [12]. Thus, having data on the exact volume of carbon emissions for a particular year might facilitate the implementation of many measures to reduce carbon levels in the energy sector.

Numerous recent studies have been undertaken to predict energy consumption. Diverse approaches, including hybrid algorithms, machine learning, and time series analysis, are employed to forecast energy consumption. The study by Ref. [13] used energy consumption data from various building types to forecast residential sector consumption. In 2021 [14], a hybrid methodology was employed to predict power consumption in Ghana. Historical data on energy consumption was utilized to guide their forecasts. Three-time series methodologies were used to predict India's energy consumption [15]. Algorithms were employed to calculate the gas and crude oil required for power generation. According to Ref. [16], researchers collected data on energy usage from 1983 to 2003 to project urban energy consumption over one year. The researchers employed fuzzy wavelet techniques to forecast electricity consumption in densely populated urban regions with substantial power demands. In 2009, research by Ref. [17] used artificial neural networks (ANN) to forecast energy consumption in Turkey up to 2020. The analysis explored differences in GDP and energy consumption across four sectors. Numerous investigations have employed autoregressive integrated moving averages and SVR techniques to forecast Turkey's upcoming energy consumption. The prediction was derived from demographic data, GDP figures, energy import/export statistics, and consumption patterns [18]. The study [19] employed a multilayer ANN methodology to forecast monthly electricity consumption in Iran over 131 months. A model was created by Ref. [20] utilizing energy consumption data from 1990 to 2006 and employing the partial least squares regression method to forecast energy consumption in the Chinese transport sector for 2010, 2016, and 2020. The LSTM algorithm was utilized by Ref. [21] to predict energy consumption, demonstrating accuracy compared to earlier studies. Independent research employs machine learning methodologies to predict energy consumption in residential areas. The study [22] introduced a technique that amalgamates machine learning algorithms, yielding enhanced and more accurate results compared to the use of algorithms in isolation. The study utilized energy usage and climate change data [23] to forecast future demand in the residential and commercial sectors. The study [24] employed data on GDP, population, energy consumption, and CO_2_ emissions as variables to predict CO_2_ emissions and energy consumption in the transportation sector, utilizing ANNs and support vector machine techniques. A case study examining electricity use in Iran's agriculture sector from 1981 to 2005 illustrates the use of GA and ANN to predict energy usage [25]. The study extensively uses LSTM technology to forecast energy usage [26]. A comparative investigation of several algorithms has definitively demonstrated that LSTM surpasses other methods in prediction accuracy. In Ref. [27], the annual cost of electricity usage in residential buildings is forecasted using ANNs and GAs. This research additionally forecasts carbon emission concentration by multi-objective optimization. The daily electricity use was predicted [28] using ARIMA, SARIMA, and LSTM algorithms to alleviate power disruptions and analyze consumption trends. Data from 1967 to 2009 was used by Ref. [3] to forecast energy consumption in the United States and Iran. The study utilized the ANN method and improved particle swarm optimization to predict power consumption from 2010 to 2030. Numerous approaches are often employed to address optimization challenges. To improve the effectiveness of energy consumption forecasting [29], employed the discrete grey model. The investigation [30] suggested utilizing the grey wolf optimizer approach to tackle an economic challenge. The study in Ref. [31] introduces a hybrid model to predict CO_2_ emissions and energy consumption within the transportation sector.

The previous section analyzed three distinct literary genres. One kind of work utilizes data sets and analytical techniques to forecast energy use. The secondary categorization of the literature employs diverse machine-learning methodologies to forecast energy use. The literature employs machine learning approaches with optimum algorithms to forecast energy usage, constituting the third category. A survey of the pertinent literature indicates that several studies endeavor to forecast total energy consumption, while others concentrate on projecting it within specific sectors. Nonetheless, the existing research lacks information concerning carbon emissions across various sectors. However, to achieve a pollution-free environment, it is imperative to anticipate energy consumption and accurately assess the levels of CO_2_ emissions. Before determining energy consumption, it is essential to ascertain energy generation. Overcoming the losses inside the system is vital to meeting the desired energy consumption. Furthermore, the prior work employed several machine learning algorithms to predict energy consumption, although it did not include feature selection to identify the most significant attributes. This study utilizes a GA-optimized SVR model to predict energy generation in Bangladesh. It also predicts energy usage in four key sectors: residential, commercial, industrial, and agricultural. Furthermore, it evaluates the carbon emissions associated with each sector.

This article organizes its subsequent sections as follows: Section Two elucidates the methodology segment. Section Three delineates the results and discussion segment. Section Four covers model validation. Section Five presents the conclusion and policy implications.

## 2 Methodology

### 2.1 Data segmentation

This dataset encompasses a comprehensive data collection on Bangladesh's power system from 1973 to 2021. It contains detailed yearly records providing insights into the country's energy infrastructure and economic indicators. Specimens were gathered from sources [[32], [33], [34], [35]]. Data on population, total GDP, total imports, total exports, net generation, and energy consumption across four sectors—residential, industrial, agricultural, and commercial—were collected annually. Net generation and population data from 1973 to 2021 and the energy consumption values for the four sectors from 2010 to 2021 were sourced from the Power Development Board (PDB) [32]. The contribution of each sector has been determined by multiplying its percentage contribution by the overall energy consumption for the year [35]. The energy consumption values for the four sectors from 2000 to 2009 were collected from the power system master plan 2016 [36]. The data regarding Bangladesh's total imports and exports from 1973 to 2021 has been sourced from reference [34,35]. Sector-specific socioeconomic consumption indicators, including year, population, total GDP, total imports, and total exports, serve as initial distinguishing factors to estimate electricity production. Following this, the filter approach [37] for feature selection is employed to pinpoint the most relevant characteristics. This filtering method assesses each feature individually using statistical metrics, ranking them based on a score derived from the correlation between each feature and the target attribute. Normalization is conducted before data partitioning to standardize features with varying range values on a uniform scale. This study evaluates the effectiveness of the regression model through k-fold cross-validation (CV). In k-fold cross-validation, the dataset is divided into k-folds, each containing approximately the same number of observations. The number of folds in this process determines the ratio of training sets to validation sets. According to Ref. [38], k-fold cross-validation designates k-1 folds as the training set while reserving the remaining data for validation. This study aims to determine the optimal number of folds for cross-validation by analyzing three performance metrics: RMSE, MAE, and the R^2^ value. The optimal fold number improves the R^2^ value while decreasing RMSE and MAE, thereby balancing training and validation set size variations.

### 2.2 SVR algorithm and its internal components

Machine learning models are ideal for accurate and reliable predictions across varied applications due to their scalability, precision, and ability to adapt to complex data patterns automatically [39]. Regression analysis frequently employs SVR, a particular category of machine learning methods under the support vector machine paradigm [40]. SVR is a machine-learning methodology grounded in the principles of statistical learning theory [41]. This study selected the SVR model owing to its unique advantages, enhancing its efficacy in various applications. Firstly, it adeptly manages both linear and non-linear data by utilizing kernel functions, ensuring reliable performance across diverse datasets [42, 43]. Secondly, SVR utilizes regularization to alleviate overfitting, which is particularly beneficial in domains with limited datasets. This enables it to function dependably despite constrained sample sizes [43]. Consequently, it addresses the issue of the random forest algorithm's inability to predict on new data [44]. Thirdly, the adaptability to employ kernels such as Gaussian and polynomial renders SVR exceptionally successful in domains like environmental and energy modelling. It surpasses conventional linear models by effectively capturing intricate, non-linear patterns [45]. The main aim of SVR is to transform the training data into a high-dimensional feature space using a nonlinear mapping function [46]. Subsequently, A regression function is employed to characterize the non-linear connection between input and output variables. The regression function outlined in equation (1) [47] is defined by a set of data ${\left(t_{j}R_{j}\right)}_{j=1}^{N}$, where $t_{j}$ denotes the input vector, $R_{j}$ indicates the actual value, and N signifies the total number of data patterns.

(1) $$Z=w\varphi \left(t_{j}\right)+b$$

The input feature is represented as φ(t), whereas the coefficients are indicated by w and b. The formula (2) [40] presented delineates the regularized risk function intended for minimization to ascertain the values of w and b.

(2) $$F\left(Z\right)=C\frac{1}{N}\sum_{j=1}^{N}L_{\varepsilon }\left(Z_{j}R_{j}\right)+\frac{1}{2}{\left\|w\right\|}^{2}$$

where,

$$L_{\varepsilon }\left(Z_{j}R_{j}\right)=\left\{ \begin{array}{c} 0,if\left|R_{j}-Z_{j}\right|\leq \varepsilon \\ \left|R_{j}-Z_{j}\right|-\varepsilon ,otherwise \end{array}\right.$$

The hyperparameters C and ε are specified, allowing precise control over the model's tuning and regularization for enhanced performance. The parameter ε represents the permissible margin of error, denoting the discrepancy between projected outcomes from the regression function and the actual observed values. The model establishes a margin encompassing all the data points within a minimal range. This discrepancy can construct a tube bounding the regression function. The dots positioned outside the tube are thought to signify training errors. The function $L_{\varepsilon }\left(Z_{j}R_{j}\right)$ in Equation (2) [40] is designated as an ε-insensitive loss function. The loss is zero if the anticipated value lies within the ε-tube. The flatness of a function may be computed using the formula $\frac{1}{2}{\left\|w\right\|}^{2}$, which represents the second part of Equation (2) [40]. Parameter C regulates the equilibrium between the model's smoothness and the empirical risk. Reduced values of variable C will provide functions that are simple or devoid of complexity, whereas elevated values may lead to overfitting of the training input data [47].

Choosing suitable kernels provides the adaptability necessary to capture intricate, nonlinear interactions in data, hence improving model performance by customizing transformations to particular data structures [48,49]. The selection of kernels facilitates the use of diverse feature spaces without explicit calculation, thereby enhancing computational efficiency in high-dimensional data processing [50]. Selecting the appropriate kernels for SVR is crucial following the identification of optimal features [51].

### 2.3 Optimization algorithms and hyper-parametric section

Evolutionary methods like GA and particle swarm optimization (PSO) for multi-objective optimization have gained popularity in machine learning due to their effectiveness in resolving conflicting objectives in complex, high-dimensional challenges [52,53]. This study conducts a multi-objective optimization of SVR parameters (C, epsilon, gamma, and kernel) utilizing GA and PSO.

#### 2.3.1 Genetic Algorithms (GA)

Genetic Algorithms are proficient in navigating extensive search spaces and circumventing local minima, rendering them beneficial for intricate, multimodal challenges. Genetic algorithms may maximize many objective functions and are readily adaptable across multiple domains, including engineering and artificial intelligence [43]. The population-based architecture of GA facilitates natural parallelization, enabling its deployment on parallel processing systems for expedited convergence [45]. It is a powerful adaptive meta-heuristic optimization technique based on the principle of natural selection. GA is often employed to improve the hyperparameters of complex machine-learning models [54]. The GA optimization process typically starts with a population of randomly generated solutions that evolve over several generations to ascertain the optimum or near-optimal solution [55]. The optimization in GAs relies on three primary operations: selection, crossover, and mutation [56]. Each individual's fitness in the population is initially evaluated based on a problem-specific fitness function [57,58]. The algorithm selects the parents using a roulette wheel selection method, then applies crossover and mutation to generate offspring with higher fitness levels [56]. After several rounds, the process produces individuals with elevated fitness, indicating whether they are the best candidates for the problem [56]. Each of these rounds is referred to as a generation. The process continues until specific termination conditions are met. If these criteria are satisfied, the process stops; otherwise, it keeps running until it finds the optimal solution or the best possible approximation [59].

#### 2.3.2 Particle swarm optimization (PSO)

PSO is lauded for its simple architecture and ease of implementation, rendering it suitable for many optimization problems [60]. Owing to its social and cognitive learning elements, PSO attains rapid convergence, resulting in effective solutions within a reduced time span [61]. The PSO [62] algorithm utilizes swarm intelligence, a global search technique. It is influenced by research on artificial life, namely birds' swarming and migratory behaviors, in their quest for sustenance. PSO emulates this behavior by modelling the collective movement of birds and modifying their trajectories depending on individual experiences and the information disseminated by the entire swarm. This collaborative method enables the algorithm to efficiently investigate various solutions, thereby increasing the probability of achieving the best result [56]. This nature-inspired swarm intelligence algorithm functions through iterative processes. It begins with a group of candidate solutions, particles in a swarm, where each particle symbolizes a possible solution. In every iteration, the positions and velocities of the particles are adjusted based on two key factors: their personal best position and the global best position. Personal best is the highest position achieved by an individual particle. In contrast, the global best is the best position identified by any particle in the entire swarm during the search process. These updates gradually enable the particles to converge toward optimal solutions [63].

#### 2.3.3 Hyperparameter tuning using optimization algorithms

Fine-tuning the hyperparameters of the SVR model is essential for achieving accurate modelling outcomes. The objective is to identify parameters that minimize the estimation error for generalization. This paper concentrates on three primary hyperparameters: the width ϵ, which controls the tolerance margin for errors; the regularization factor C, which applies penalties for violations of constraints; and σ, a parameter that defines the width of the Gaussian Radial Basis Function's (RBF) kernel. Every parameter enhances the model's capacity to generalize unseen data effectively. The investigation enhances the hyperparameters by applying GA and PSO. Both optimization strategies utilize the cross-validated SVR as their objective function. The optimal values of the hyperparameters yield a high R^2^ score while minimizing RMSE and MAE. The optimal optimization approach yields the best results relative to alternatives. An ideal, optimization-based, cross-validated SVR model achieves power generation forecasting. In this research, the initial population was generated randomly. First, the hyperparameter range was defined, and then the initial population for the optimization algorithm was created within those predefined boundaries.

### 2.4 Model development

#### 2.4.1 Sectoral prediction of energy consumption

**Fig. 1:**
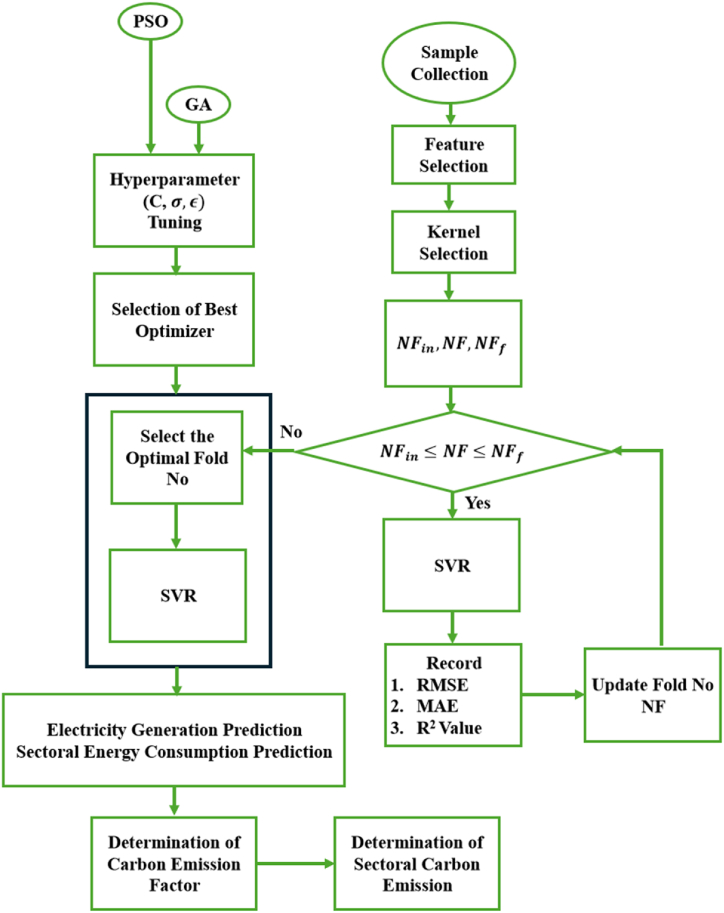
Proposed model of GA-optimized SVR. Where NF is the number of folds, ${NF}_{in}$ is the initial fold number and ${NF}_{f}$ is the final fold number.

The proposed model initially utilizes year, population, GDP, imports, and exports as input variables. Subsequently, optimal features are selected using a feature selection method. These optimal features are then used as input variables in a GA-optimized SVR model to predict electricity generation. After determining electricity generation, this value and the optimal features are used as inputs to predict energy consumption in the domestic, utilizing a GA-optimized SVR model. A cross-validated SVR model with manually calibrated parameters evaluates energy consumption in the industrial sectors. Initial parameters are approximated based on the tuning outcomes from a GA-optimized SVR model previously employed to forecast energy consumption in the residential sector. Manual modifications are selected to shorten computation time and improve model speed. Following an assessment of consumption in both the domestic and industrial sectors, this data serves as input alongside optimal inputs to train a GA-optimized SVR model for predicting energy consumption in the commercial sector. Subsequently, after estimating energy consumption in the domestic, industrial, and commercial sectors, this data is used to predict energy consumption in the agricultural sector, once again employing distinct GA-optimized SVR models. The different stages of model development are represented in Fig. 1.

#### 2.4.2 Determination of sector-wise carbon emission

The 2021 daily report of the Power Grid Company of Bangladesh (PGCB) is the source for data collection to evaluate the energy supply from various fuel types [64]. The subsequent equations [65] determine the carbon footprint linked to each fuel source.

(3) $${GEF}_{Fuel,GHG}=\frac{{EF}_{Fuel,GHG}}{\eta_{Fuel}}$$

(4) $$E_{Fuel,GHG}\left(t\right)={G_{Fuel}\left(t\right)\times GEF}_{Fuel,GHG}$$

(5) $$E_{Fuel,GHG}^{{CO}_{2}-e}\left(t\right)=E_{Fuel,GHG}\left(t\right)\times {GWP}_{GHG}$$

$$Here,{GWP}_{GHG}=1\;for\;{CO}_{2},25\;for\;{CH}_{4\;},and\;298\;for\;N_{2}O\text{,}$$

(6) $$E_{Fuel}^{{CO}_{2}-e}\left(t\right)=\sum_{GHGs}E_{Fuel,GHG}^{{CO}_{2}-e}\left(t\right)$$

(7) $$E_{Total}^{{CO}_{2}-e}\left(t\right)=\sum_{Fuel}E_{Fuel}^{{CO}_{2}-e}\left(t\right)$$

(8) $$G_{Total}\left(t\right)=\sum_{Fuel}{GEF}_{Fuel}$$

(9) $$CI\left(t\right)=\frac{E_{Total}^{{CO}_{2}-e}\left(t\right)\;}{G_{Total}\left(t\right)}$$

This analysis assesses emission levels by examining the three leading greenhouse gases: CO_2_, CH_4_, and N_2_O. Due to Bangladesh's lack of a national greenhouse gas inventory, emission factors specific to fuel from the IPCC's Fourth Assessment Report (AR4) are employed to compute ${\text{GEF}}_{\text{Fuel},\text{GHG}}$ [41]. Equations (3), (4), (5), (6), (7), (8), (9) [65] calculated a fuel's carbon emissions in 2021. The fuel's emission factor per watt-hour is calculated by dividing its total carbon emissions by the energy produced, as stated in equation (10) [66]. Table 1 presents the emission factors for various fuels, as established by the analysis conducted in 2021.

(10) $$E_{factor}^{f_{t}}=\frac{{TC}_{s}^{f_{t}}}{{TE}_{E}^{f_{t}}}$$

**Table 1:** The 2021 emission factors [64].

Types of fuels	Energy Supply (TWh)	Carbon Emission (10^9^ g of CO_2_ Equivalent)	Emission Factor/Wh
HSD	0.1737	146.69	0.8440
Coal	4.7210	4895.00	1.0368
Oil	19.9530	12133.00	0.6081
Natural Gas	48.2130	23704.10	0.4917
Carbon-neutral sources	7.9743	0	0

$E_{factor}^{f_{t}}$ = Fuel emission factor per watt-hour $f_{t}$.

$f_{t}$ = Fuel type like Gas, HSD, coal, or oil.

${TC}_{s}^{f_{t}}$ = Total fuel carbon emissions $f_{t}$.

${TE}_{E}^{f_{t}}$ = Total fuel energy $f_{t}$.

The average emission factor is determined by taking the mean of the emission factors for all fuels analyzed in 2021, resulting in a value of 0.745165. For simplicity, this study calculates the carbon emissions for each subsequent year using this fixed emission factor. Equation (11) [66] can estimate carbon emissions for various sectors.

(11) $${CE}_{Sector}=E_{s}^{Sector}\times 0.745165$$

${CE}_{Sector}$ = Carbon emissions by different sectors

$E_{s}^{Sector}$ = Sector energy demand. The sectors may include agriculture, housing, industry, and commerce.

## 3 Result and discussion

**Fig. 2:**
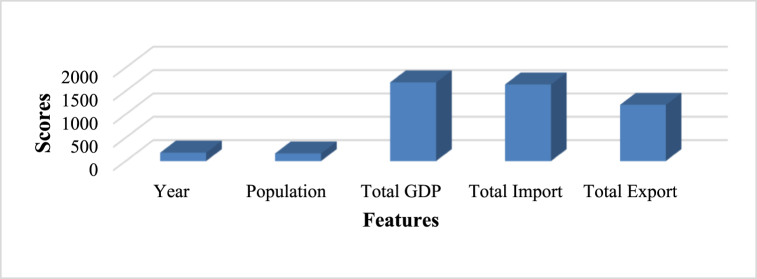
Represents the feature's significance.

This research proposes an optimal prediction model to estimate energy consumption across four sectors—domestic, industrial, agricultural, and commercial. In addition to predicting energy consumption, the study calculates carbon emissions generated by these sectors. The process begins with forecasting power generation, using inputs such as population, total GDP, total imports, and total exports to predict power generation for a given year. The F-test approach is applied for feature selection to identify the optimal characteristics. Using the F-test approach reveals that GDP, exports, and imports score higher than the year and population characteristics, as shown in Fig. 2. These three features—GDP, imports, and exports—are sufficient for identifying data patterns. In contrast, the year and population variables are inconsequential, meaning their exclusion will not affect the outcome. Thus, the three primary characteristics used to train the SVR model for predicting energy generation are GDP, imports, and exports.

**Fig. 3:**
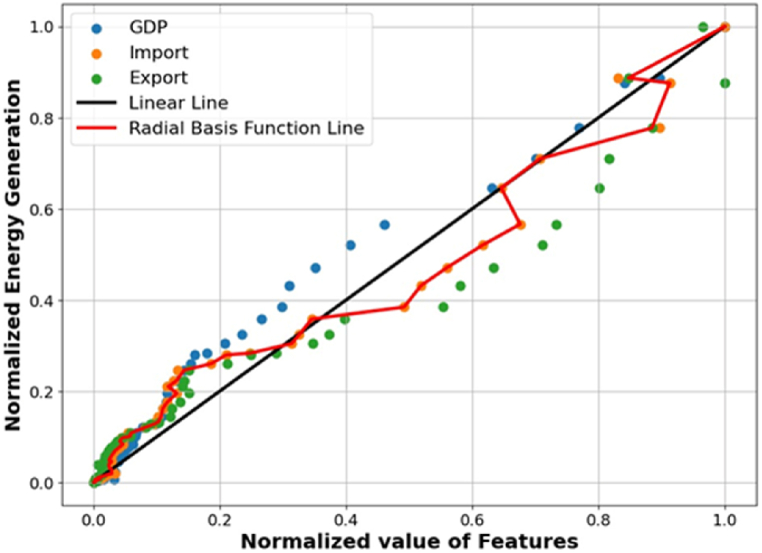
Selection of kernel for SVR.

Fig. 3 illustrates the process of selecting kernels for SVR by plotting three selected characteristics against overall power generation to identify the most suitable kernels that align closely with the data. Two distinct types of kernels are represented: the black line corresponds to the linear kernel function, while the red line represents the radial basis kernel function. This figure shows that most data points are positioned significantly from the diagonal line. The red line intersects with most data points, suggesting a strong alignment with the radial basis function. In the SVR model, the radial basis function exhibits superior generalization capacity compared to the linear function, positioning it as the optimal kernel for precise energy generation forecasting. However, despite this selection, the SVR model does not demonstrate consistent or reliable performance. Training the model on 80 % of the data and evaluating it on the remaining 20 % limits its ability to comprehend the attributes of the complete dataset, particularly when the training data is chosen randomly. The partitioning of the constrained training dataset into separate training, validation, and testing subsets markedly affects the precision of the training procedure. This study utilizes k-fold cross-validation to augment the model's predicted accuracy and enhance its dependability with new data. Selecting a suitable fold threshold is crucial for attaining the best outcomes using SVR. The quantity of folds chosen in cross-validation affects the training set's magnitude. Determining the optimal number of folds necessitates assessing the SVR model across a range of fold counts, often between 2 and 24. The ideal number of folds is established by evaluating three distinct metrics: RMSE, MAE, and the R^2^ value. The optimal fold count will produce a high R^2^ value while reducing RMSE and MAE.

**Fig. 4:**
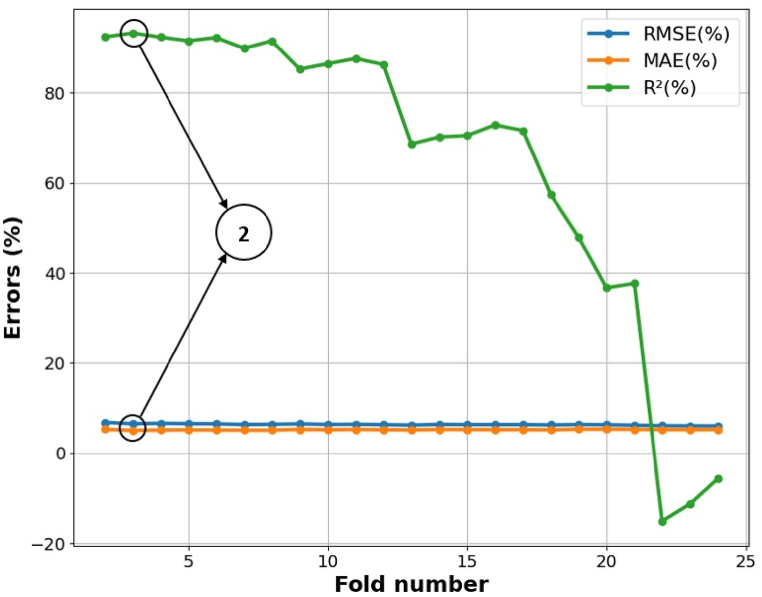
Selection of optimal fold number.

**Fig. 5:**
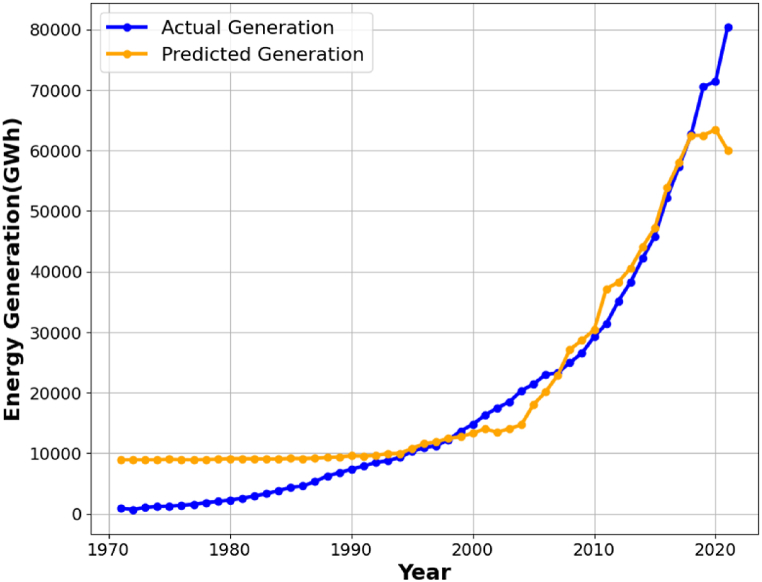
Represents the actual and predicted value of energy generation using cross-validated SVR.

**Fig. 6:**
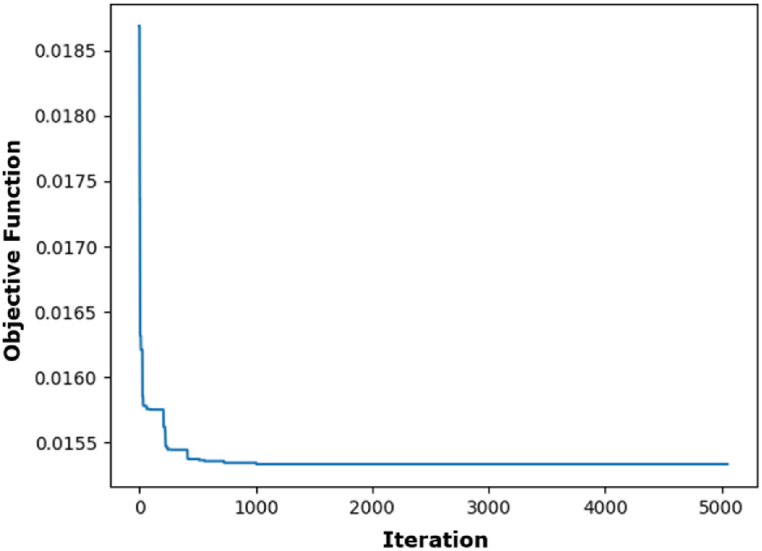
Optimization process of SVR using GA.

**Fig. 7:**
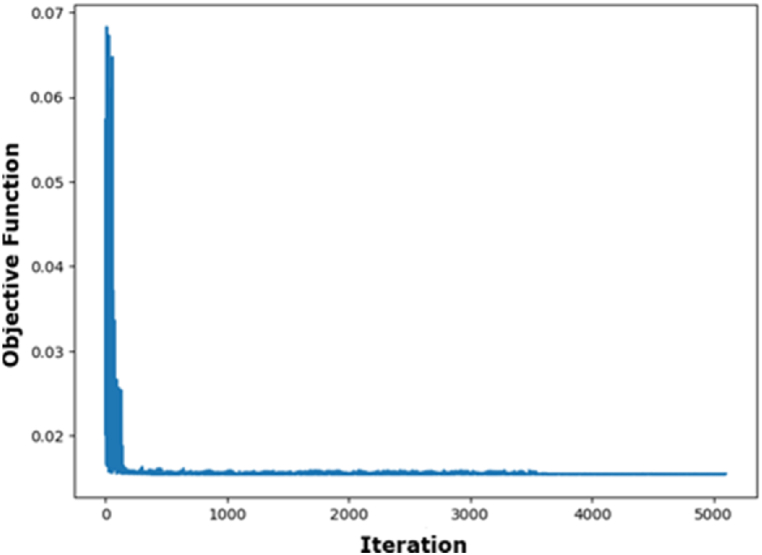
Optimization process of SVR using PSO.

Fig. 4 illustrates that the RMSE and MAE errors maintain relative stability with increased fold numbers. However, the R^2^ value diminishes as the fold number escalates. According to the data in Fig. 4, a fold number of 3 is optimal, as it preserves a comparatively high R^2^ value while exhibiting markedly decreased RMSE and MAE values. Fig. 5 illustrates the actual and projected energy consumption values generated by the optimally cross-validated SVR model, highlighting a significant difference between observed and predicted energy usage. The cross-validated SVR model yields an RMSE of 6.50 %, an MAE of 5.02 %, and an R^2^ value of 93.29 %, which is considered inadequate. GA and PSO are used to fine-tune the hyperparameters of the SVR model, enhancing its performance. The GA and PSO optimization processes are represented in Fig. 6, Fig. 7, respectively. Both optimization algorithms attain low objective function values; however, GA demonstrates a more consistent and stable final convergence than PSO, which exhibits minor variations even after achieving a low goal value. It suggests that GA are oriented towards attaining a more stable solution, while PSO continues to explore the area surrounding the optimal point.

**Fig. 8:**
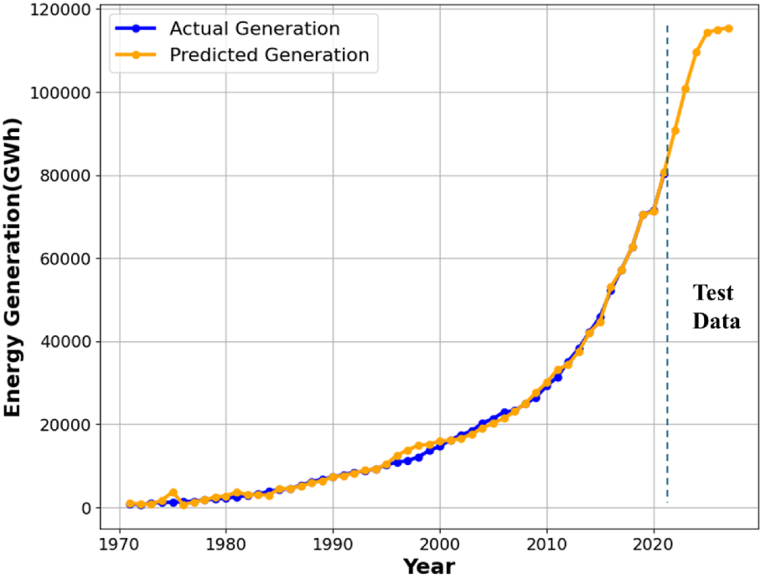
Prediction of energy generation using cross-validated SVR optimized with GA up to 2027.

It also delineates the errors arising from tuning using the two optimization strategies. Table 2 also demonstrates that the SVR model optimized with GA surpasses the model optimized with PSO. Thus, the SVR model augmented by GA is the superior option for predicting energy use. Precise forecasting of future electricity generation necessitates data on total imports and exports. An improved SVR model has been developed to mitigate the scarcity of this data. This model uses GDP and population to generate total exports and imports as outputs. The SVR model employs a linear kernel to project total import and export values until 2027, considering the direct correlation between population and GDP. Table 3 presents the figures for imports and exports. The GA-optimized SVR model uses these factors to predict energy generation until 2027, as seen in Fig. 8.

**Table 2:** Represents the Errors and hyperparameters of SVR.

	Parameters	Initial Ranges	Optimization Methods	
			SVR with PSO	SVR with GA
Hyperparameters of SVR	C	0–100	98.951	83.197
	Epsilon	0.001–1	0.0017	0.0016
	Gamma	0.001–1	0.573	0.6003
	Kernel	Liner, Polynomial, Radial Basis Function	Radial Basis Function	Radial Basis Function
Errors	% RMSE		1.54	1.53
	% MAE		1.19	1.18
	% R^2^		99.62	99.63

**Table 3:** Estimated values of total import and export.

‵ Years	Total Import (Billion US $)	Total Export (Billion US $)
2022	75.50	47.61
2023	82.83	51.06
2024	89.01	54.93
2025	96.96	59.19
2026	104.69	63.36
2027	111.93	67.77

Fig. 8 illustrates the projected energy usage using a SVR model optimized by a GA, demonstrating a robust correlation between the predicted and actual energy consumption. The GA-SVR model achieves an RMSE of 1.53 % and MAE of 1.18 %, indicating considerable precision. Moreover, the model's impressive R^2^ score of 99.63 % ensures that predictions closely correspond with actual data. A clear correlation exists between the increase in energy output and the growth of GDP, imports, and exports. The forecast for the energy generation curve shows a plateau in 2027, indicating a slowdown in the growth of imports and exports compared to previous data trends.

**Fig. 9:**
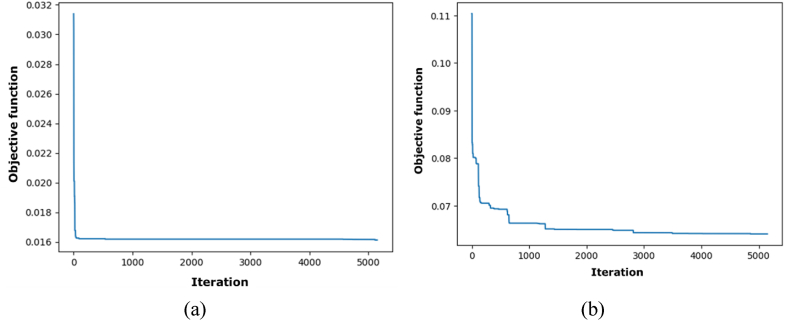
Optimization process of SVR using GA for (a) domestic sector and (b) agricultural sector.

Fig. 9(a) indicates a swift decrease in the objective function for the domestic sector, ultimately stabilizing at approximately 0.016 following the initial iterations. The objective function rapidly decreases and maintains a relatively stable value after around 2000 iterations. Fig. 9(b) demonstrates a progressive decline in the objective function for the agricultural sector, commencing from a higher value. The value stabilizes at approximately 0.05, exhibiting fluctuations before convergence after the iterations. The rapid convergence of the optimization process to a stable value in both sectors indicates its efficacy. The values of the hyperparameters of SVR for various sectors are presented in Table 4.

**Table 4:** Hyperparameters of SVR for different sectors.

Sectors	Hyperparameters			
	C Range: 0-100	Gamma Range: 0.001–1	Epsilon Range: 0.001–1	Kernel Range: Linear, Polynomial, Radial Basis Function
Domestic	95.57	0.2699	0.0067	Linear
Agricultural	99.45	0.4202	0.0011	Radial Basis Function
Industrial	83.19	0.004	0.01	Radial Basis Function
Commercial	50.33	0.767	0.023	Linear

**Fig. 10:**
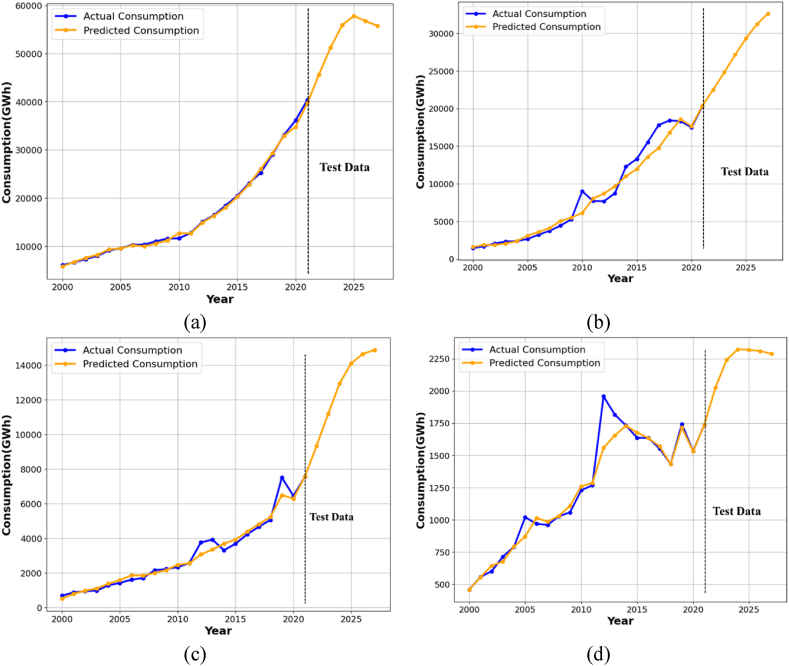
Actual vs predicted plot of (a) domestic sector, (b) industrial sector, (c) commercial sector, and (d) agricultural sector.

Fig. 10 compares each sector's actual and expected energy consumption, revealing a negligible disparity between the two figures. This slight inconsistency ensures enhanced predictive capability. The proposed predictive model estimates domestic, industrial, agricultural, and commercial energy consumption, with RMSE values of 1.61 %, 6.54 %, 6.40 %, and 5.75 %, respectively.

**Fig. 11:**
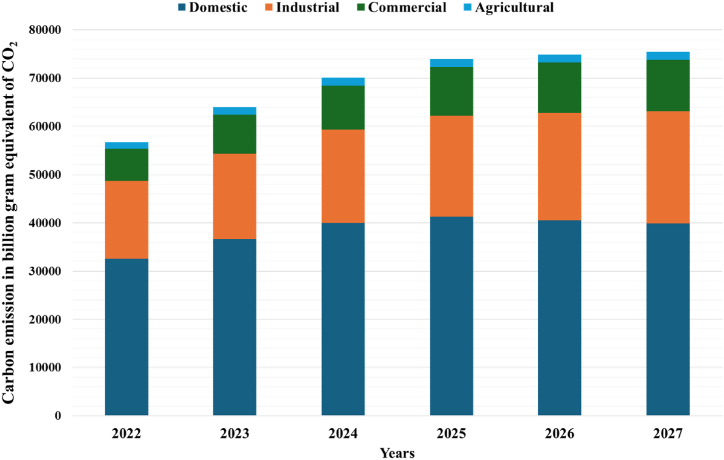
Represents the carbon emissions from different sectors from 2022 to 2027.

The R^2^ values for these sectors are 99.64 % for residential, 96.71 % for industrial, 95.65 % for commercial, and 94.73 % for agricultural. The average absolute error values for the domestic, industrial, commercial, and agricultural sectors are 1.22 %, 4.98 %, 4.40 %, and 4.04 %, respectively. Projected energy consumption in 2027 is estimated to be 55748.66 GWh for the domestic sector, 32642.35 GWh for the industrial sector, 14892.49 GWh for the commercial sector, and 2288.37 GWh for the agricultural sector. Additionally, the residential sector remains Bangladesh's primary energy consumer. From 2020 to 2027, energy consumption is projected to increase by around 54 % in the residential sector, 86 % in the industrial sector, and 33 % in agricultural sector, relative to 2020. Although household consumption constitutes the predominant share of energy consumption, the industrial sector in Bangladesh is exhibiting a more rapid development rate than other sectors, attributable to the nation's progressing position. Evaluating energy usage and carbon output across various sectors is crucial for attaining a sustainable economy. This analysis forecasts energy consumption across sectors and calculates the associated carbon emissions, as illustrated in Fig. 11. Projections indicate that the four sectors are expected to emit 74008.56, 74934.01, and 75437.96-billion-gram equivalent carbon in the years 2025, 2026, and 2027, respectively. The majority of emissions originate from the household and industrial sectors. To effectively reduce carbon emissions, it is essential to incorporate zero-carbon sources within the energy sector to mitigate emissions.

A crucial aspect of regression diagnostics involves analyzing the characteristics of residuals, which can assist in uncovering any shortcomings in the model. The regression model's assumptions indicate that the residuals are random variables that adhere to a normal distribution, exhibit independence, possess a zero mean, and maintain a constant variance. The variance remains constant if the residuals exhibit a normally distributed random variable with a zero mean. Visual depiction is a useful tool for identifying outliers in residuals. The model is deemed viable if the residuals exhibit random fluctuations around a zero-mean graph. Assume the residuals are symmetrically distributed around the line with a zero mean. If such is the case, it may be indicative of homoscedasticity. Homoscedasticity is a distribution characterized by a constant variance across all observed or predicted values. An instance of heteroscedastic variance is a residual distribution whose magnitude diverges from the expected or actual values. Such phenomena are termed heteroscedastic variance. The residual distribution displays a fan-shaped pattern, with the size of the residuals either growing or decreasing about the magnitude of the projected or actual values.

**Fig. 12:**
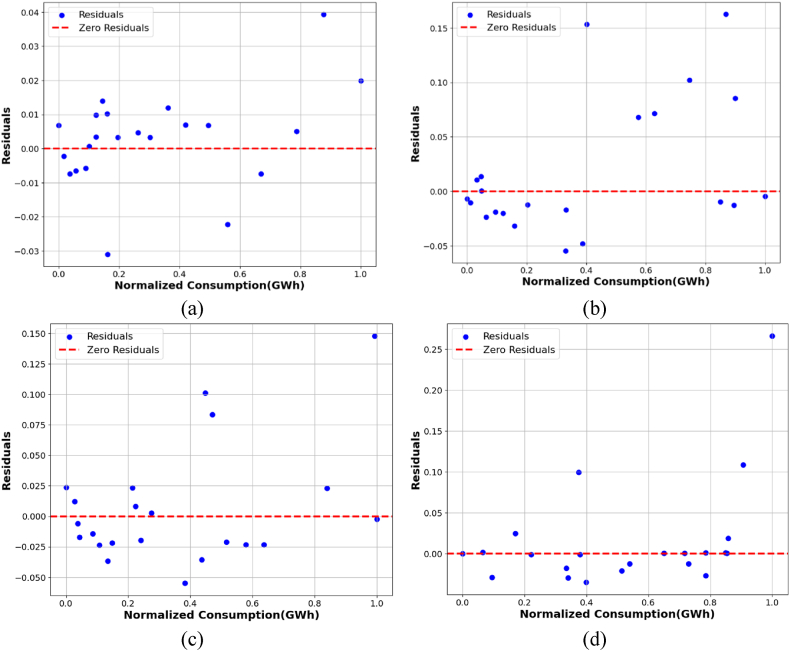
Residuals plot for (a) domestic sector, (b) industrial sector, (c) commercial sector and (d) agricultural sector.

This study identifies residuals using the residual plot depicted in Fig. 12. The graphic indicates that the residuals are uniformly distributed around their mean of zero. Furthermore, the residuals are independent and exhibit no identifiable patterns. Thus, it can be concluded that the residuals of the proposed model demonstrate homoscedasticity. Homoscedasticity obstructs any positive relationship between variance and RMSE, thereby hindering an increase in both variance and RMSE. Standardized or studentized residuals are also effective for identifying residuals that have considerable divergence from the mean. Residuals are generally considered unusual when they exceed ±2 standard deviations [67]. The standard deviations for the residuals across the domestic, commercial, industrial, and agricultural sectors are 0.014, 0.050, 0.060, and 0.073, respectively. The residual plot for these four sectors demonstrates that most points fall within the range of ±2 standard deviations. Therefore, the residual diagnostics validate the model's reliability and bolster the validity of the proposed methodology.

### 3.1 Sensitivity analysis

The sensitivity of an algorithm denotes the degree to which the model's output fluctuates in reaction to a particular alteration in an input value. It elucidates the impact of various inputs on the outcomes. The investigation is essential for comprehending how models respond to variations in input values, noise resilience, data integrity, internal configuration, and additional variables. This research employed the one-at-a-time (OAT) technique [68] to evaluate the sensitivity of the suggested model. During model assessment, OAT methodologies alter a single input at a time while keeping all other inputs fixed. Most OAT approaches typically begin with a predetermined set of inputs that serve as the basis for the model's convergence. If the model's evaluation of a particular set of baseline values changes with any modification of variables, then the modified input is accountable for the variance. The research investigates the impact of four variables—GDP, total imports, total exports, and net generation—on the sensitivity of the output.

To assess the impact of variations in GDP, total imports, total exports, and net generation on output, each variable is adjusted by increasing or decreasing it by 5 %–50 % from its original value. According to equation (12) [69] the relative sensitivity index (RSI) is found by comparing the root mean square error (RMSE) caused by an input's change to the RMSE caused by the input's baseline composition.

(12) $$\text{Relative}\;\text{Sensitivity}\;\text{Index}=\frac{{\text{RMSE}}_{\text{after}\;\text{variation}}}{{\text{RMSE}}_{\text{base}\;\text{line}}}$$

**Fig. 13:**
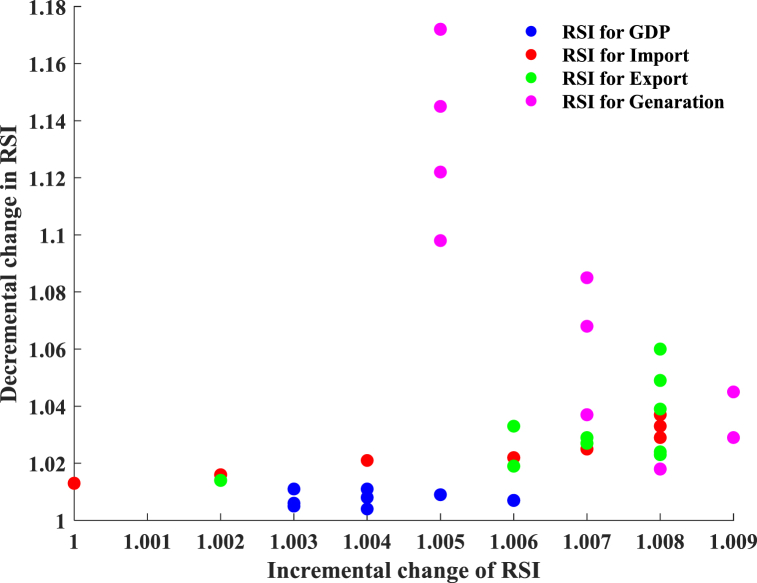
Sensitivity analysis for the domestic sector.

Fig. 13 illustrates the sensitivity analysis for the domestic sector, indicating that for all input features, the rise in the Relative Sensitivity Index (RSI) due to a reduction in input values from the baseline surpasses the impact of a similar increase. Nonetheless, these differences are relatively insignificant. The average increase in RSI for positive changes in GDP, total imports, total exports, and net generation is 0.4 %, 0.3 %, 0.6 %, and 0.8 %, respectively. The average decline in RSI due to reductions in GDP, total imports, total exports, and net generation shows a notable difference, with recorded figures of 0.9 %, 1.7 %, 2 %, and 3.2 %, respectively. The findings indicate that RSI experiences a more pronounced impact within the domestic sector when input values decrease by 5 %–20 % from their baseline levels. The input feature net generation shows a notable comprehension of its influence on RSI. The rate of change in RSI shows a notable increase when input values are reduced by 30 %–50 % from the baseline.

**Fig. 14:**
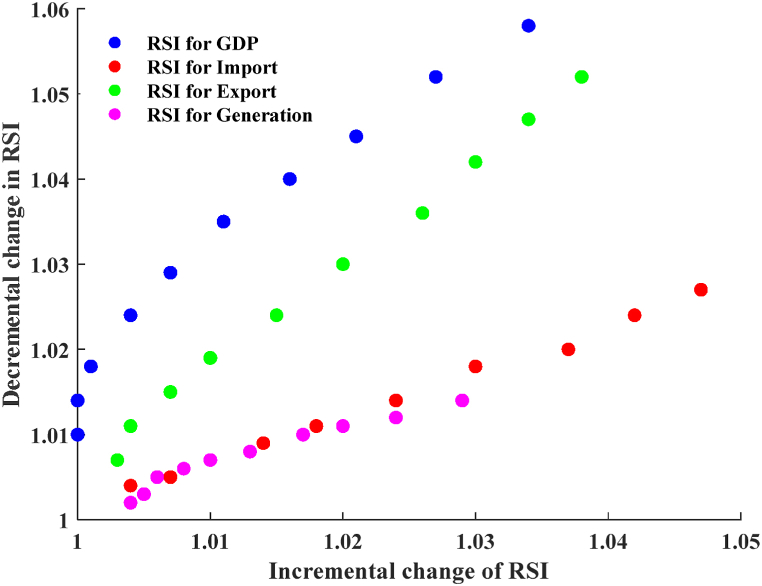
Sensitivity analysis for the agricultural sector.

Fig. 14 illustrates the sensitivity analysis of the agriculture sector. The graphic depicts a linear relationship between the incremental and decremental changes in input characteristics on RSI. As the percentage variation of the input characteristics from the baseline value escalates, the RSI rises from the baseline RSI. When the characteristics of the inputs go up by 5 %–20 % from the starting point, the average RSI values for GDP, imports, exports, and generation go up by 0.13 %, 0.86 %, 0.6 %, and 0.6 %, respectively. Taking away between 5 % and 20 % of the characteristics of the inputs from their starting point leads to average RSI values of 1.6 % for GDP, 0.63 % for imports, 1.3 % for exports, and 0.4 % for generation. It is evident that when the input feature is increased, the export is highly responsive to the output, while when the input feature is decreased, the GDP is extremely sensitive to the output. When input values decrease by 30 %–50 % from the baseline, the RSI change rate significantly increases.

**Fig. 15:**
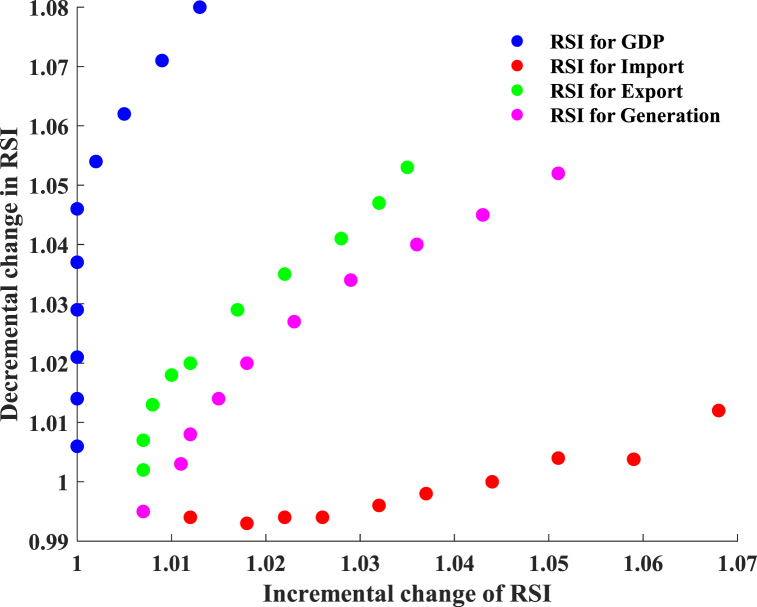
Sensitivity analysis for the commercial sector.

Commercial sector sensitivity analysis is shown in Fig. 15. Average RSI values for GDP, imports, exports, and generation climb by 0 %, 1.95 %, 0.8 %, and 1.12 % when input characteristics increase by 5 %–20 % from baseline. Average RSI values for GDP, imports, exports, and generation are 1.75 %, 0 %, 1 %, and 0.5 %, respectively, after reducing 5 %–20 % of input characteristics. Increased input characteristics enhance import responsiveness to output, whereas decreased input features raise GDP sensitivity to production. GDP growth and a decrease in imports have little effect on output.

**Fig. 16:**
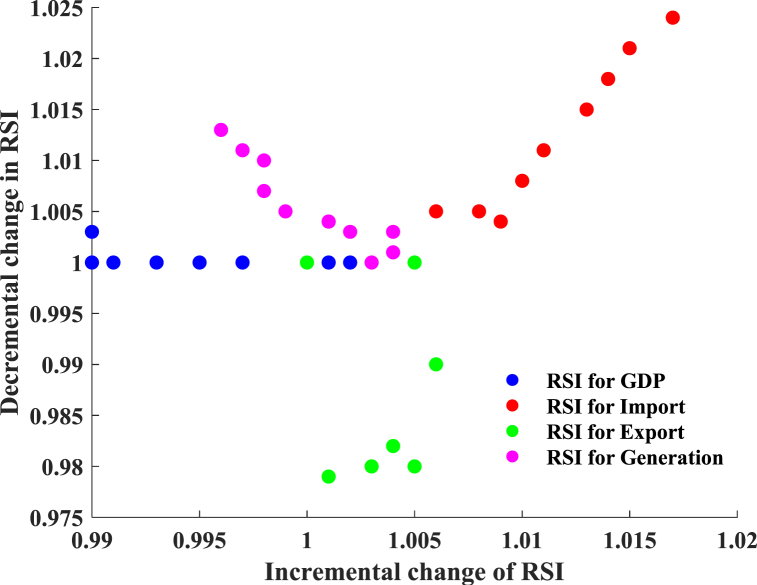
Sensitivity analysis for the industrial sector.

Fig. 16 illustrates the sensitivity analysis of the industrial sector. The average RSI values for GDP, imports, exports, and generation increase by 0.2 %, 0.74 %, 0.16 %, and 0.28 %, respectively, when input characteristics increase by 5 %–20 % from the baseline. The average RSI values for GDP, imports, exports, and generation are 0 %, 0.5 %, 0 %, and 0.22 %, respectively, after reducing 5 %–20 % in input characteristics. Altered input features enhance the responsiveness of imports to outputs. Fig. 16 demonstrates that an increase in the input from 25 % to 50 % results in linear growth of the RSI, indicating a significant sensitivity to the output. After adjusting the input from 25 % to 50 %, the RSI derived from the change in exports decreased from one. Altering the export and import characteristics by more than 25 % from their baseline values is inadvisable.

## 4 Model validation

**Fig. 17:**
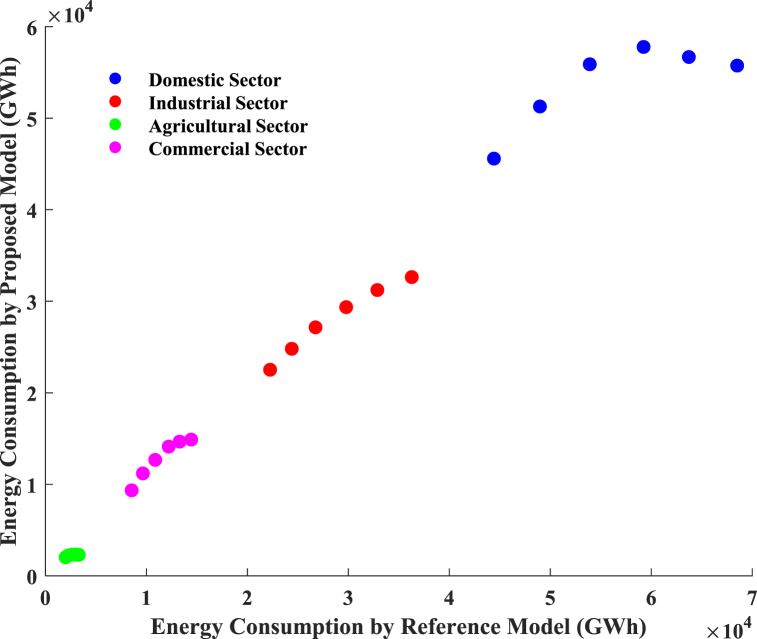
Represents the validation of the proposed model.

The proposed model is corroborated by a comparison with the reference model detailed in the research article [70]. In Ref. [70], the author employed the Long-range Energy Alternative Planning (LEAP) model to analyze Bangladesh's electricity sector from 2022 to 2041. To estimate Bangladesh's energy requirements, four scenarios were examined: low growth (LG), medium growth (MG), high growth (HG), and business as usual (BAU). In LEAP, the demand side was divided into five categories: residential, commercial, industrial, agricultural, and others. Various scenarios from 2022 to 2041 predicted Bangladesh's energy needs. The projected GDP growth rates for the BAU, LG, MG, and HG scenarios are 8.95 %, 6.9 %, 15.8 %, and 18.4 %, respectively. This investigation employs GDP data to develop the model under high-growth scenarios. The results of the proposed model are thus compared with the outcomes of the reference model's high growth scenarios illustrated in Fig. 17. It demonstrates that both models' energy consumption across the four sectors exhibits a linear correlation. The two results show a minor distinction in the industrial, domestic, and commercial sectors. A noticeable difference occurred in the domestic sector between 2026 and 2027. The reference model focuses solely on the constant growth rate of GDP, while the proposed model incorporates a broader range of input features, including total imports, exports, generation, and GDP. In contrast, the proposed model incorporates a broader range of input features, including total import, export, generation, and GDP. Fig. 8 demonstrates that the net generation for 2026 and 2027 remained stable since each year's import and export figures exhibited little fluctuations compared to previous years. As a result, the results of the proposed model may differ somewhat from those of the reference model.

## 5 Conclusion and policy implications

A crucial tool for energy management and demand response in industrialized and developing nations is the accurate prediction of energy output and consumption by governments and decision-makers. This study outlines a methodology for forecasting energy usage in four major sectors of Bangladesh: residential, industrial, commercial, and agricultural. This study utilizes a GA-optimized SVR model to predict electricity generation and energy consumption in different sectors. The research further assesses carbon emissions across several sectors. This research employs several approaches to create a reliable prediction model. The F-test approach is initially utilized to determine the best qualities, encompassing GDP, imports, and exports. Cross-validation is used to enhance the efficacy of the SVR model, and the optimal number of folds for cross-validation is identified. The optimal number of cross-validated folds is established as 3. The hyperparameters of SVR are optimized using GA and PSO to enhance the prediction model's performance. The SVR model, when enhanced with GA, surpasses the PSO technique. The research demonstrates that when optimized using GA, SVR effectively predicts electricity generation, with an RMSE of 1.61 %. The model's projection anticipates energy output to attain 115,421 GWh by 2027. The suggested model precisely predicts the energy consumption of four sectors: residential, commercial, industrial, and agricultural. The Mean Absolute Error (MAE) for each sector is as follows: 1.22 % for residential, 4.40 % for commercial, 4.98 % for industrial, and 4.04 % for agricultural. The survey indicated that the residential sector is the predominant energy user in Bangladesh. However, the industrial sector is experiencing a notable rise in energy consumption. The model forecasts that energy consumption in residential, industrial, agricultural, and commercial sectors will increase by 54 %, 86 %, 49 %, and 4 %, respectively, from 2020 to 2027. In 2025, 2026, and 2027, these four industries will emit 74008.56, 74934.01, and 75437.96-billion grams of carbon, respectively. The analysis reveals that the residential and industrial sectors mainly contribute to most emissions. The analysis of residuals for the proposed model indicated that the residuals demonstrate homoscedasticity. Standardized residual plots are employed to evaluate the existence of homoscedasticity in the residuals. The standard deviation for each sector is calculated, indicating that most residuals lie within ±2 standard deviations. This ensures the absence of aberrant behaviors in the residuals. One or two data points exceed ±2 standard deviations due to the abrupt change in data points for a certain industry during that year. This study performed a sensitivity analysis of the proposed model to assess the influence of different input parameters on the model's results. This study analyzes the sensitivity of each input feature across four areas of energy use. In the residential sector, net generation variability is more sensitive to output than other input factors. The variations in the input factors of imports and GDP demonstrate heightened responsiveness to the farm sector's reaction. The variation in GDP also affects the performance of the business sector, and the variability of input feature imports affects the industrial sector's output. This study validates the suggested model by comparing its results with those of reference models. Although the proposed model's results deviate somewhat from the reference model's, the outcomes generally closely resemble those of the reference model. This study assesses energy consumption and carbon emissions across various sectors. However, it does not analyze the different carbon emission mitigation scenarios. Future research will evaluate mitigation strategies and identify potential sources and techniques to meet emission reduction targets.

The results of the proposed model suggest several important policy implications, outlined below. These insights will provide a foundation for informed decision-making, guiding initiatives that may lead to significant changes in the specified areas.

1. **Strategic Planning and Allocation of Resources in the Energy Sector**: Accurate energy consumption predictions across residential, industrial, commercial, and agricultural sectors enable more precise energy resource allocation. By utilizing the model's specified input parameters, specifically total GDP, imports, and exports, the energy consumption of the four primary sectors for any upcoming year can be determined. This analysis indicates that the anticipated energy consumption in 2027 is projected to be 55748.66 GWh for the residential sector, 32642.35 GWh for the industrial sector, 14892.49 GWh for the commercial sector, and 2288.37 GWh for the agricultural sector. These insights assist policymakers in optimizing infrastructure investments, ensuring energy security, and enhancing capacity planning to meet future demand adequately.
2. **Carbon Emission Reduction Strategies**: The model's capacity to predict sector-specific carbon emissions enables policymakers to formulate tailored emission reduction plans for specific sectors. This analysis predicted that in 2027, these four sectors, namely residential, commercial, industrial, and agricultural, will emit 75437.96-billion-gram carbon equivalents. It was also discovered that the residential and industrial sectors are the leading sources of carbon emissions. Consequently, it will assist policymakers in implementing effective carbon reduction measures, such as promoting renewable energy sources, energy-efficient appliances, and green construction standards, by focusing on the domestic and industrial sectors.
3. **Renewable Energy Integration**: Given the expected rise in energy consumption, integrating renewable energy sources into the electricity generation mix is crucial. This study, due to its ability to predict future energy consumption and emissions, can aid policymakers in choosing renewable energy infrastructure to meet increasing demand while reducing carbon emissions, particularly in high-consumption sectors.
4. **Long-Term Energy and Environmental Planning**: The study's projections of energy use and carbon emissions until 2027 can assist in formulating long-term strategies for energy and sustainability. This allows the government to establish achievable targets for energy production, consumption, and emissions that align with national development objectives and international climate commitments, including the Paris Agreement.

## CRediT authorship contribution statement

**Md. Sadikul Hasan:** Writing – original draft, Visualization, Validation, Software, Resources, Methodology, Investigation, Formal analysis, Data curation. **Md. Tarequzzaman:** Writing – review & editing, Writing – original draft, Visualization, Validation, Supervision, Software, Resources, Methodology, Investigation, Formal analysis, Data curation, Conceptualization. **Md. Moznuzzaman:** Visualization, Validation, Resources, Investigation. **Md Abdul Ahad Juel:** Visualization, Validation, Resources.

## Submission declaration

• I declare that the work described has not been published previously except in the form of an academic thesis.
• I declare that the article is not under consideration for publication elsewhere.
• I declare that the article's publication is approved by all authors and tacitly or explicitly by the responsible authorities where the work was carried out.
• I declare that if accepted, the article will not be published elsewhere in the same form, in English or in any other language, including electronically without the written consent of the copyright-holder.

## Declaration of competing interest

The authors declare that they have no known competing financial interests or personal relationships that could have appeared to influence the work reported in this paper.
